# Implication of changes in xanthine oxidase activity following hemodialysis

**DOI:** 10.1186/s12882-023-03062-z

**Published:** 2023-01-16

**Authors:** Hayato Fujioka, Tsutomu Koike, Teruhiko Imamura, Kota Kakeshita, Hidenori Yamazaki, Koichiro Kinugawa

**Affiliations:** The Second Department of Internal Medicine, Toyama University, Toyama, 930-0194 Japan

**Keywords:** Xanthine oxidase, Hemodialysis, Hemodynamics, Uremia

## Abstract

**Background:**

Xanthine oxidase activity has a key role in the development of oxidative stress and progression of cardiovascular diseases. However, the change of xanthine oxidase activity following hemodialysis and its prognostic impact remain uncertain.

**Methods:**

We prospectively included hemodialysis patients who did not take any anti-hyperuricemic agents and measured their xanthine oxidase activity before and after the index hemodialysis. The impact of change in xanthine oxidase activity during hemodialysis on cardiovascular death were investigated.

**Results:**

A total of 46 patients (median 72 years old, 29 men) were included. During hemodialysis, a common logarithm of xanthine oxidase activity decreased significantly from 1.16 (0.94, 1.27) to 1.03 (0.80, 1.20) (*p* < 0.01). Of them, xanthine oxidase activity remained unchanged or increased in 16 patients, who had a greater decrease in blood pressure and more hemoconcentration compared with others. Two–year survival from cardiovascular death was not significantly stratified by the changes in xanthine oxidase activity (*p* = 0.43).

**Conclusions:**

During hemodialysis, xanthine oxidase activity decreased among the overall cohort, whereas some patients experienced its increases, which might be associated with hypotension and hemoconcentration during hemodialysis. Further larger-scale studies are required to validate our findings and find clinical implication of change in xanthine oxidase activity during hemodialysis.

## Background

Xanthine oxidase has a principal role in the oxidative stress system, which facilitates organ reperfusion injury, endothelial dysfunction, systemic hypertension, and heart failure [1, 2]. Xanthine oxidase is activated via tissue hypoxia and inflammatory cytokines [2]. Xanthine oxidase is disseminated systematically beyond the ischemic organ [3].

Xanthine oxidase activity in patients receiving hemodialysis due to end-stage renal disease remains poorly understood. Some authors reported elevated xanthine oxidase activity among them [4–6], whereas its association with other clinical parameters as well as its causality have not yet been well clarified. We, in this study, investigated the change in xanthine oxidase activity following hemodialysis and its prognostic implication.

## Methods

### Patient selection

Patients with end-stage renal diseases who received hemodialysis for over 3 months at our institute or associated institutes were prospectively included as a concern cohort. Patients taking anti-hyperuricemic agents including allopurinol, febuxostat, and topiroxostat were excluded. Healthy volunteers without any specific comorbidities who took medical tests at our institute or associated institutes were also included as a control cohort.

Plasma xanthine oxidase activity was measured in all participants as detailed below. The study was approved by the local institutional ethical review board beforehand. All participants received written informed consent before enrollment.

### Xanthine oxidase activity measurement

For all hemodialysis patients, their plasma samples were obtained just before the index hemodialysis on beginning of the week to measure xanthine oxidase activity by using a high-performance liquid chromatography method [7]. The obtained plasma were centrifuged within one hour and reacted with pterin solution for three hours. During the reaction, pterin was converted to isoxanthopterin by xanthine oxidase. Converted isoxanthopterin was quantified by fluorometric detector. The amount of isoxanthopterin that was synthesized per hour was defined as xanthine oxidase activity. Xanthine oxidase activity was measured just after the index hemodialysis in the same manner.

In a healthy cohort, plasma xanthine oxidase activity was measured in the same manner from the plasma obtained at the index medical tests. All measured xanthine oxidase activity data were converted to common logarithm.

### Other collected data

Baseline characteristics including demographics, comorbidity, and laboratory data were obtained. In a concern cohort, changes in vital sign and laboratory data following the hemodialysis were obtained. Interdialytic weight gain rate was calculated as follows: [(pre-dialysis weight [kg]) – (post-dialysis weight (of the prior session) [kg])] / (dry weight [kg] × 100). Kt/V level for urea was calculated by the Daugirdas method. Serum calcium level was adjusted as follows, when serum calcium level was less than 4.0: actual serum calcium [mg/dL] + [4.0 – (serum albumin [g/dL])]. Geriatric nutritional risk index was evaluated as nutrition status and calculated as follows: [14.89 × serum albumin (g/dL)] + {41.7 × [current body weight (kg)/standard body weight (kg)]}, in which standard body weight was calculated as [height (m)]^2^× 22 [8].

### Statistical analysis

Continuous variables were expressed as median and interquartile and compared between the two groups using Mann–Whitney U test. Wilcoxon signed-rank test was performed to compare the coupled data such as common logarithm of xanthine oxidase activity before and after hemodialysis. Categorical variables were expressed as numbers and percentages and compared between the two groups using Fisher’s exact test.

A change in xanthine oxidase activity during hemodialysis was a primary concern. Association between xanthine oxidase activity and other clinical parameters as well as clinical outcomes was a secondary concern.

The association between baseline xanthine oxidase activity and other clinical characteristics was assessed by linear regression analysis. We investigated two-year all-cause death and cardiovascular death between the xanthine oxidase non-decreased group and the decreased group.

In all analyses, 2-tailed *p* < 0.05 was considered statistically significant. Analyses were performed using R software version 3.5.2 (R Foundation for Statistical Computing, Vienna, Austria).

## Results

### Baseline characteristics

A total of 46 patients were included (Table 1). The median age was 72 (66, 83) years old and 29 were men. The median baseline xanthine oxidase activity was 14.30 (8.80, 18.43) nmol/L/hr and its common logarithm was 1.16 (0.95, 1.27).

**Table 1:** Basic clinical and biochemical data of patients

Factor	*N* = 46
Demographics	
Age (years)	72 (66, 83)
Men (number, %)	29 (63)
Hemodialysis vintage (years)	5.7 (3.0, 12.6)
Dry weight (kg)	54.3 (47.6, 61.1)
Interdialytic weight gain rate (%)	4.5 (3.7, 4.8)
Systolic blood pressure (mmHg)	150 (133, 164)
Diastolic blood pressure (mmHg)	74 (67, 84)
Kt/V for urea	1.51 (1.37, 1.71)
Cardio-thoracic ratio (%)	52.2 (48.6, 54.9)
Diabetes mellitus (number, %)	31 (67)
History of cardiovascular disease (number, %)	26 (55)
Medications	
Angiotensin receptor blocker	15 (32)
Diuretics	5 (11)
Statin	13 (28)
Etilefrine / amezinium metilsulfate	17 (37)
Laboratory data	
Hemoglobin (g/dL)	10.4 (9.9, 11.2)
Aspartate aminotransferase (IU/L)	10 (9, 14)
Alanine aminotransferase (IU/L)	8 (6, 11)
γ-glutamyl transpeptidase (IU/L)	21 (16, 30)
Serum albumin (g/dL)	3.1 (3.0, 3.3)
Serum urea nitrogen (mg/dL)	59.9 (50.5, 70.0)
Serum creatinine (mg/dL)	9.8 (8.7, 11.0)
Serum uric acid (mg/dL)	7.0 (6.4, 7.9)
Serum sodium (mEq/L)	140 (139, 142)
Serum potassium (mEq/L)	4.5 (4.0, 5.0)
Serum adjusted calcium (mg/dL)	9.1 (8.6, 9.7)
Serum phosphorus (mg/dL)	4.9 (4.3, 5.5)
Serum bicarbonate (mmol/L)	21.2 (20.2, 23.2)
Serum low-density lipoprotein cholesterol (mg/dL)	80 (65, 106)
Serum triglyceride (mg/dL)	109 (69, 162)
Serum C-reactive protein (mg/dL)	0.20 (0.10, 0.47)
Geriatric nutritional risk index	86.3 (81.8, 89.3)
Baseline XO (nmol/L/hr)	14.30 (8.80, 18.43)
Baseline log_10_XO	1.16 (0.95, 1.27)

Continuous variables are presented as median and interquartile. Categorical variables are presented as number and percentage

*XO* xanthine oxidase

**Fig. 1:**
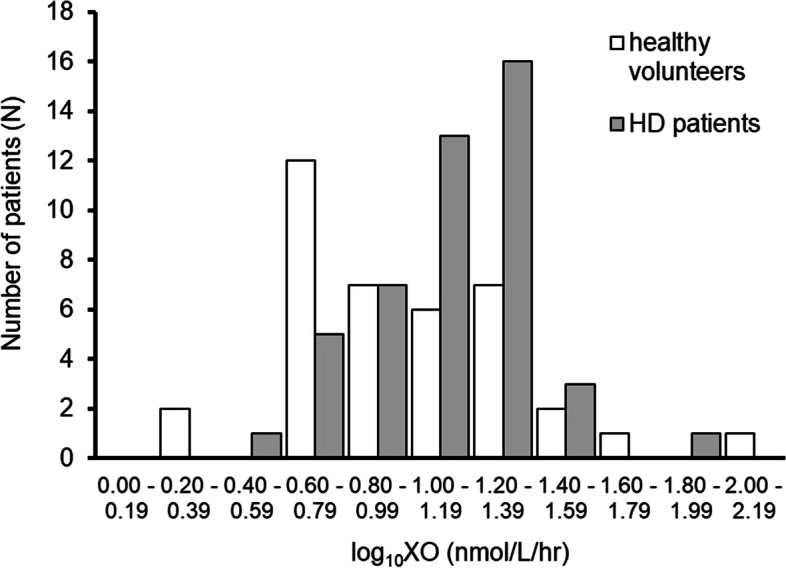
Distribution of log_10_XO in healthy volunteers and HD Patients. HD; hemodialysis, XO; xanthine oxidase.

The median of the common logarithm of xanthine oxidase activity of 38 healthy volunteers was 0.90 (0.70, 1.25), which was significantly lower than the present cohort (*p* < 0.005). The distribution of baseline xanthine oxidase activity among the two cohorts is displayed in Fig. 1.

### Baseline xanthine oxidase activity and other parameters

Among baseline characteristics, Kt/V for urea, asparate aminotransferase, alanine aminotransferase, γ-glutamyl transpeptidase, serum sodium, and bicarbonate were significantly correlated with the common logarithm of xanthine oxidase activity (*p* < 0.05 for all, Table 2). Of them, alanine aminotransferase was independently associated with the common logarithm of xanthine oxidase activity (*p* < 0.001, Table 2).

**Table 2:** Association between xanthine oxidase activity before hemodialysis and other clinical parameters

	**Univariate Analysis**		**Multivariate Analysis**		
	**Beta value**	***p* value**	**Beta value**	***p* value**	**VIF**
Age (years)	0.057	0.7			
Male	5.77	0.14			
Hemodialysis vintage (years)	-0.26	0.33			
Interdialytic weight gain rate (%)	-2.52	0.14			
Systolic blood pressure (mmHg)	0.095	0.26			
Kt/V for urea	-13.83	< 0.05^*^	-3.49	0.34	1.101
Diabetes mellitus	0.043	0.59			
History of cardiovascular disease	2.00	0.61			
Hemoglobin (g/dL)	2.56	0.057			
Aspartate aminotransferase (IU/L)	1.3	< 0.001^*^			
Alanine aminotransferase (IU/L)	0.94	< 0.001^*^	0.9	< 0.001^*^	1.342
γ-glutamyl transpeptidase (IU/L)	0.26	< 0.001^*^			
Serum albumin (g/dL)	2.1	0.71			
Serum urea nitrogen (mg/dL)	0.13	0.32			
Serum creatinine (mg/dL)	0.88	0.28			
Serum uric acid (mg/dL)	2.71	0.15			
Serum sodium (mEq/L)	-1.42	< 0.01^*^	-0.38	0.19	1.203
Serum potassium (mEq/L)	-0.54	0.7			
Serum adjusted calcium (mg/dL)	-0.61	0.82			
Serum phosphorus (mg/dL)	-0.54	0.7			
Serum bicarbonate (mmol/L)	-2.05	< 0.05^*^	0.28	0.57	1.265
Serum C-reactive protein (mg/dL)	1.88	0.32			
Geriatric nutrition risk index	0.26	0.42			

Variables that are considered clinically potential confounders were included in the multivariate analysis after excluding their multicollinearity with VIF < 5.0. Multivariate R^2^-adjusted = 0.601, *p* < 0.001

^*^p < 0.05 by linear regression analysis

### Changes in xanthine oxidase activity during hemodialysis

**Fig. 2:**
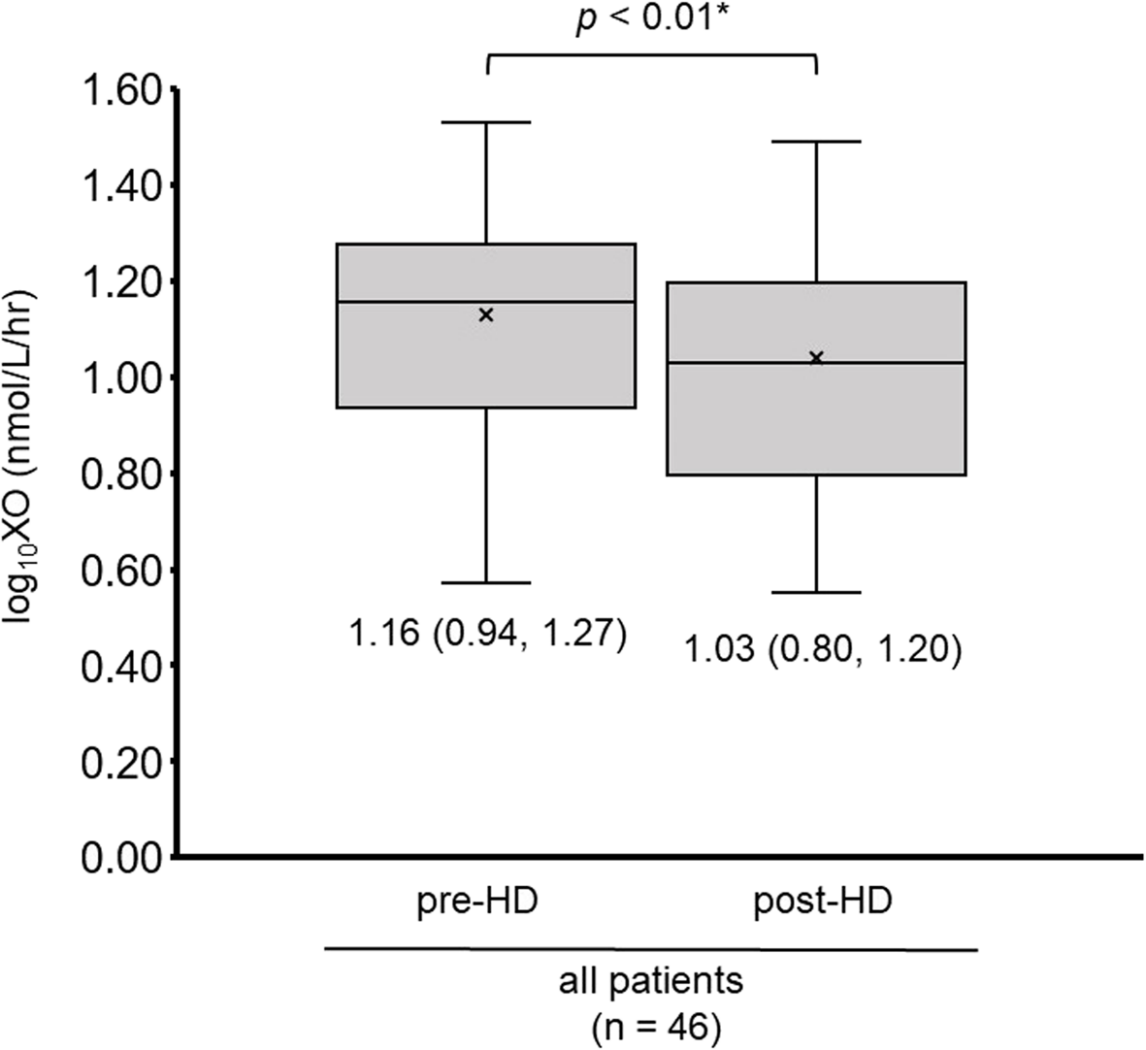
Changes in plasma xanthine oxidase activity during dialysis in all patients. HD; hemodialysis, XO; xanthine oxidase. *p < 0.05.

Among all the hemodialysis cohort, the common logarithm of xanthine oxidase activity decreased significantly during hemodialysis [from 1.16 (0.94, 1.27) to 1.03 (0.80, 1.20), *p* < 0.01; Fig. 2].

**Fig. 3:**
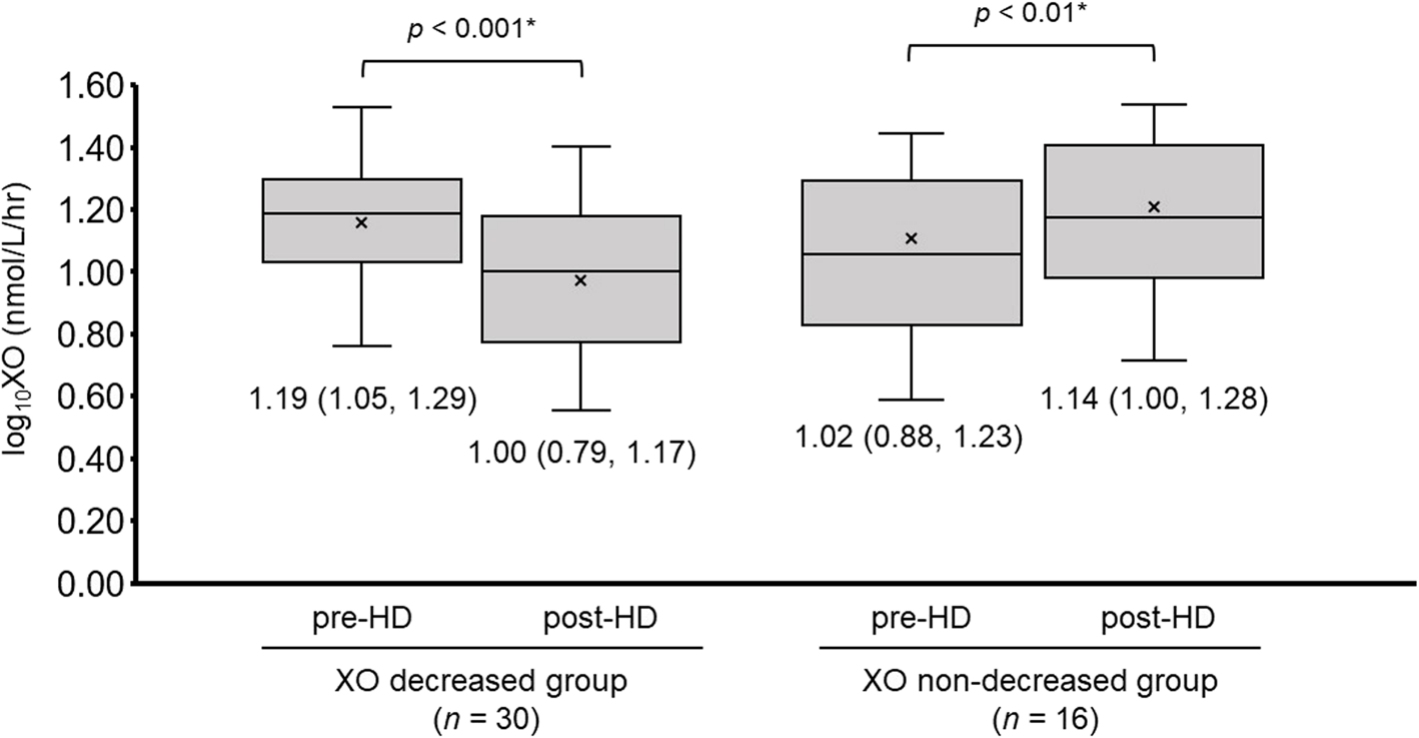
Changes in plasma xanthine oxidase activity during hemodialysis in XO decreased group or XO non-decreased group. HD; hemodialysis, XO; xanthine oxidase. *p < 0.05.

Xanthine oxidase activity levels decreased in 30 patients (decreased group) whereas remained unchanged or rather increased in the residual 16 patients (non-decreased group) (Fig. 3). At baseline, xanthine oxidase activity levels were not significantly different between the two groups [1.19 (1.05, 1.29) versus 1.02 (0.88, 1.23), *p* = 0.15]. Following hemodialysis, xanthine oxidase activity levels decreased in the decreased group whereas increased in the non-decreased group (*p* < 0.01 for both). As a result, post-dialytic xanthine oxidase activity level was higher in the non-decrease group [1.00 (0.79, 1.17) versus 1.14 (1.00, 1.28), *p* < 0.08].

### Characteristics stratified by the changes in xanthine oxidase activity

Baseline characteristics were compared between the two groups (non-decreased versus decreased groups) (Table 3). There were no significant differences in basic characteristics between the two groups, whereas serum urea nitrogen, uric acid and bicarbonate tended to be higher in the non-decreased group (*p* ≤ 0.10 for all).

**Table 3:** Baseline characteristics compared between XO non-decreased group and XO decreased group

Factor	XO non-decreased group (*n* = 16)	XO decreased group (*n* = 30)	*p*
Demographics			
Age (years)	68 (61, 79)	74 (67, 83)	0.27
Men (number, %)	10 (63%)	19 (63%)	1.00
Hemodialysis vintage (years)	9.7 (2.8, 12.7)	4.7 (2.9, 11.3)	0.43
Dry weight (kg)	55.6 (49.4, 64.8)	53.1 (47.1, 58.3)	0.32
Interdialytic weight gain rate (%)	4.0 (3.7, 4.8)	4.5 (3.7, 4.8)	0.76
Systolic blood pressure (mmHg)	151 (132 181)	149 (135, 164)	0.75
Diastolic blood pressure (mmHg)	81 (70, 85)	73 (67, 81)	0.28
Kt/V for urea	1.44 (1.26, 1.59)	1.51 (1.40, 1.79)	0.13
Diabetes mellitus	11 (69%)	20 (67%)	1.00
History of cardiovascular disease	10 (63%)	16 (53%)	0.76
Medications			
Etilefrine / amezinium metilsulfate (number, %)	7 (44%)	10 (33%)	0.53
Laboratory data			
Hemoglobin (g/dL)	10.5 (10.1, 11.3)	10.3 (9.7, 11.1)	0.36
Aspartate aminotransferase (IU/L)	10 (9, 13)	10 (9, 15)	0.67
Alanine aminotransferase (IU/L)	9 (8, 13)	8 (6, 11)	0.12
γ-glutamyl transpeptidase (IU/L)	21 (18, 24)	20 (14, 33)	0.57
Serum albumin (g/dL)	3.2 (2.9, 3.4)	3.1 (3.0, 3.3)	0.75
Serum urea nitrogen (mg/dL)	65.2 (56.9, 73.5)	57.9 (47.6, 67.2)	0.10
Serum creatinine (mg/dL)	10.1 (8.6, 12.6)	9.7 (8.7, 10.8)	0.32
Serum uric acid (mg/dL)	7.4 (7.0, 7.9)	6.7 (6.2, 7.7)	0.06
Serum sodium (mEq/L)	141 (139, 142)	140 (138, 142)	0.75
Serum potassium (mEq/L)	4.6 (4.3, 5.0)	4.5 (4.0, 5.1)	0.60
Serum adjusted calcium (mg/dL)	9.0 (8.7, 9.8)	9.2 (8.6, 9.6)	0.87
Serum phosphorus (mg/dL)	5.1 (4.5, 6.0)	4.8 (4.3, 5.5)	0.38
Serum bicarbonate (mmol/L)	20.7 (19.8, 21.8)	22.0 (20.4, 23.5)	0.10
Serum C-reactive protein (mg/dL)	0.25 (0.12, 0.50)	0.10 (0.10, 0.38)	0.32
Geriatric nutrition risk index	87.5 (85.2, 91.1)	84.9 (81.6, 87.9)	0.22

Continuous variables are presented as median and interquartile. Categorical variables are presented as number and percentage

*XO* xanthine oxidase

Changes in clinical parameters during hemodialysis were compared between the two groups (Table 4). Patients in the non-decreased group had a greater decrease in systolic blood pressure and a greater increase in hemoglobin and albumin (*p* < 0.05 for all). Interdialytic plasma volume decrease had a trend to be lower in the non-decreased group (*p* = 0.09).

**Table 4:** Comparison of Interdialytic weight gain, changes in vital signs and blood tests before and after dialysis in the XO non-decreased and decreased groups

Factor	XO non-decreased group (*n* = 16)	XO decreased group (*n* = 30)	*p*
delta systolic blood pressure (mmHg)	-18 (-39, -6)	-3 (-17, 11)	0.03^*^
delta diastolic blood pressure (mmHg)	-4 (-19, 2)	1 (-5, 8)	0.10
delta hemoglobin (g/dL)	1.1 (0.5, 1.4)	0.3 (0.0, 0.7)	0.03^*^
delta albumin (g/dL)	0.3 (0.3, 0.6)	0.2 (0.0, 0.4)	0.04^*^
delta urea nitrogen (mg/dL)	-46.1 (-53.8, -38.8)	-42.4 (-47.1, -33.1)	0.13
delta creatinine (mg/dL)	-7.1 (-7.7, -5.6)	-6.5 (-7.2, -5.6)	0.32
delta uric acid (mg/dL)	-5.4 (-6.3, -4.9)	-5.1 (-5.7, -4.6)	0.24
delta sodium (mEq/L)	-1 (-2, 1)	1 (-1, 3)	0.08
delta potassium (mEq/L)	-1.4 (-1.6, -1.1)	-1.3 (-1.6, -1.0)	0.74
delta adjusted calcium (mg/dL)	1.1 (0.7, 1.5)	1.2 (0.7, 1.5)	0.76
delta phosphorus (mg/dL)	-2.9 (-3.9, -2.4)	-2.7 (-3.2, -2.1)	0.39
Interdialytic plasma volume decrease (%)	8.7 (5.1, 12.1)	5.4 (1.9, 8.6)	0.09

Delta “X” = ([X]_post-HD_ –[X]_pre-HD_), HD; hemodialysis

Interdialytic plasma volume decrease = (Ht_post-HD_ – Ht_pre-HD_) / Ht_post-HD_ × 100. Ht, hematocrit

*HD* hemodialysis, *XO* xanthine oxidase

^*^p < 0.05

### Prognostic impact of change in xanthine oxidase activity

**Fig. 4:**
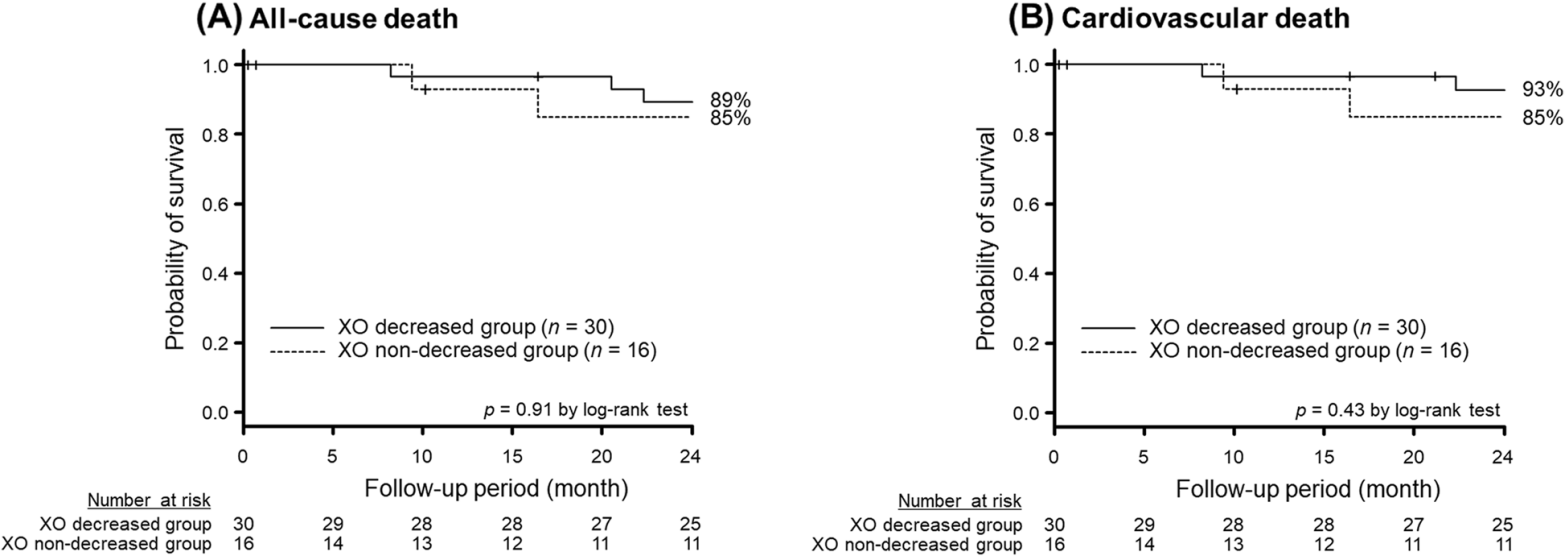
Two–year freedom from (**A**) all-cause death or (**B**) cardiovascular death stratified by the change in XO activity during hemodialysis. XO; xanthine oxidase

There were 5 deaths including 4 cardiovascular deaths during the 2-year observational period. Freedoms from all-cause death and from cardiovascular death were not significantly stratified by the change in xanthine oxidase activity (*p* = 0.91 and *p* = 0.43, respectively; Fig. 4AB).

## Discussion

In this study, we investigated the change in xanthine oxidase activity during hemodialysis and its prognostic impact. Major findings are follows: (1) Baseline xanthine oxidase activity was higher in patients with hemodialysis than the healthy cohort; (2) Xanthine oxidase activity decreased following hemodialysis among the overall cohort; (3) A non-decrease in xanthine oxidase activity during hemodialysis was associated with interdialytic decrease in blood pressure and plasma volume.

### Xanthine oxidase activity in hemodialysis patients

Consistently to our finding, several studies consistently reported the incremental xanthine oxidase activity in the hemodialysis patients [4–6]. Other studies rather observed relatively lower xanthine oxidase activity in patients with renal dysfunction independent on hemodialysis [9, 10]. Hemodialysis itself might have a considerable impact on the xanthine oxidase activity.

Kt/V for urea, which is an index of hemodialysis effectiveness, was negatively associated with baseline xanthine oxidase activity. In other words, ineffective hemodialysis was associated with incremental baseline xanthine oxidase activity, which is consistent with a previous report that uremia was associated with incremental xanthine oxidase activity [5]. Uremia-associated chronic inflammation might stimulate the synthesis and activity of xanthine oxidase [11, 12]. Patients dependent on hemodialysis, in general, have metabolic acidosis due to the loss of hydrogen ion excretion from urine. Metabolic acidosis accompanying decrease in bicarbonate might stimulate xanthine oxidase activity.

Extracellular fluid overload that results in hyponatremia might induce inflammation and incremental xanthine oxidase activity [13]. Xanthine oxidase family is stored in the liver [2]. Correlation between xanthine oxidase activity and liver enzyme, which was also observed in a healthy cohort study in another study [14], would be logical.

### Changes in xanthine oxidase activity during hemodialysis

Xanthine oxidase activity decreased following hemodialysis in the overall cohort. Choi JY and colleagues observed that incremental xanthine oxidase activity was accompanied by the hypoxia-induced metabolisms prior to the hemodialysis [5]. Given the mechanism of hemodialysis, it might be reasonable that xanthine oxidase activity decreases during hemodialysis in general due to the improvement in above-described abnormalities.

On the contrary, xanthine oxidase activity rather increased in some patients. Those with non-decreased xanthine oxidase activity had more intradialytic hypotension. Xanthine oxidase is synthesized under the hypoxic situation by hypoxia-inducible factor-1α [15, 16]. Oxidative stress might be facilitated by inappropriately excessive hemodialysis accompanying tissue ischemia.

Differently from our finding, Miric and colleagues observed incremental activity of xanthine oxidase during hemodialysis in patients with malnutrition [6]. Risk factors in increased xanthine oxidase activity during hemodialysis remain the future concerns.

### Prognostic impact of xanthine oxidase activity

A decline in blood pressure during hemodialysis is associated with mortality and morbidity [17]. Given the above discussion, a non-decrease in xanthine oxidase activity during hemodialysis might have a negative prognostic impact. Nevertheless, freedom from all-cause or cardiovascular death were not stratified by the change in xanthine oxidase activity.

We, in general, attempt our best to adjust dry weight and medications to prevent hemodynamic deterioration during and/or following hemodialysis. Such efforts might have been attempted in patients with non-decreased xanthine oxidase activity. A single assessment of xanthine oxidase activity might be insufficient to predict hard endpoint. Further studies with longer-term observational period would clarify the prognostic impact of change in xanthine oxidase activity during hemodialysis.

## Limitations

This study is a proof-of-concept including small sample size. A small event number and a short observational period would be one of the reasons why the time-to-event analyses did not reach statistical significance. We measured xanthine oxidase activity only two times per patient (before and after the index hemodialysis). The trend of xanthine oxidase activity was not assessed. We assessed only mortality as a hard endpoint in this study. The change in xanthine oxidase activity during hemodialysis might have prognostic impact on other soft endpoints including exercise capacity and quality of life.

## Conclusions

During hemodialysis, xanthine oxidase activity decreased among the overall cohort, whereas some patients experienced its increases, which might be associated with more hypotension and hemoconcentration during hemodialysis. Prognostic impact of increase in xanthine oxidase activity during hemodialysis remains the future concern.

## Data Availability

The data that support the findings of this study are available from the corresponding author upon reasonable request.
